# Long‐Term Effectiveness and Safety of Dorsal Root Ganglion Stimulation for Persistent Spinal Pain Syndrome: Results From an Expanded Prospective Registry

**DOI:** 10.1111/papr.70183

**Published:** 2026-07-01

**Authors:** Pedram Tabatabaei, Maria Eriksson, Amar Awad, Johan Vänman

**Affiliations:** ^1^ Department of Clinical Science Umeå University Umeå Sweden; ^2^ Department of Diagnostics and Intervention Umeå University Umeå Sweden

## Abstract

**Introduction:**

Persistent spinal pain syndrome (PSPS) with predominant low‐back pain (LBP) is difficult to manage when conservative and surgical treatments fail. Dorsal root ganglion stimulation (DRG‐S) offers segmental, focal neuromodulation that may be particularly suited for managing axial LBP, yet long‐term evidence from real‐world practice remains limited. This study evaluated the effectiveness, safety, and impact on medication use of bilateral T12 DRG‐S in patients with intractable LBP, using prospectively collected registry data.

**Methods:**

Of 33 consecutive patients with chronic LBP assessed for DRG‐S, 20 underwent trial stimulation after multidisciplinary evaluation. Eighteen (90%) proceeded to permanent implantation following ≥ 50% pain reduction during trial. Patients were followed via a digital platform at 3, 6, 12, and 24 months. Outcomes included pain intensity (NRS), quality of life (PROMIS‐29), pain catastrophizing (PCS), satisfaction, medication use, and adverse events. Statistical comparisons were made between baseline and follow‐up time points using paired tests.

**Results:**

Pain intensity decreased substantially and significantly across all follow‐up assessments: mean NRS declined from 8.0 ± 1.8 at baseline to 3.6 ± 1.6 at 3 months, 3.2 ± 1.8 at 6 months, 3.6 ± 1.7 at 12 months, and 2.9 ± 1.6 at 24 months (all *p* < 0.001). At 24 months, 100% of patients achieved ≥ 30% pain reduction and 70% achieved ≥ 50% reduction. Patient‐reported outcomes demonstrated broad improvements. PROMIS‐29 domains showed significant gains in physical function, pain interference, fatigue, and sleep disturbance, with consistent improvements also observed in anxiety and depression. Pain catastrophizing (PCS) scores decreased markedly, with total scores reduced by 47%–62% across follow‐ups (all *p* < 0.001), reflecting improvements across helplessness, magnification, and rumination subdomains. Medication use declined over time. Mean opioid dose fell from 27 mg/day at baseline to 11.25 mg/day at 1 year and 4.44 mg/day at 2 years (*p* = 0.019 and 0.011, respectively). Paracetamol use also decreased significantly, whereas gabapentinoid use remained variable. Seven hardware‐related complications were recorded (lead migration *n* = 3, lead fracture/damage *n* = 4), all successfully managed with revision surgery. Kaplan–Meier analysis estimated mean time to complication at 1080 days. Two patients discontinued therapy (EOT), both for reasons unrelated to device failure.

**Conclusions:**

In this prospective registry, bilateral T12 DRG‐S provided durable reduction in pain intensity, improved physical, psychosocial, and cognitive outcomes, and facilitated meaningful opioid‐sparing effects over 2 years. Complications were limited to correctable hardware issues, and therapy discontinuation was rare. Although larger multicentre studies are warranted, these findings add important real‐world evidence supporting DRG‐S as a valuable therapeutic option for refractory axial LBP.

## 1 Introduction

Chronic low back pain (LBP) remains one of the most challenging pain syndromes to treat once conservative and surgical measures fail. Dorsal root ganglion stimulation (DRG‐S) has been proposed as a promising option, but its long‐term effectiveness and safety are not well established. Given the global burden of LBP and the limitations of existing therapies, there is a critical need to explore and validate new strategies. LBP remains the leading cause of disability worldwide with 619 million people affected in 2020 according to the World Health Organization, and the prevalence is expected to rise if current trends continue [1]. Although most episodes resolve spontaneously, a substantial minority progress to chronic LBP—now classified as chronic primary LBP or persistent spinal pain syndrome (PSPS types 1–2)—that is refractory to conservative and surgical treatment [2, 3]. For this group, interventional strategies such as nerve blocks, radiofrequency ablation, and tonic spinal cord stimulation (SCS) offer only modest or short‐lived benefit, and long‐term opioid therapy carries well‐recognized risks [4, 5, 6, 7, 8, 9, 10, 11].

Dorsal root ganglion stimulation (DRG‐S) has emerged as a targeted neuromodulation technique that acts on primary sensory neurons at segmental levels closely associated with the pain focus. Early controlled trials demonstrated superior responder rates for DRG‐S compared with tonic SCS in focal neuropathic pain [12]. Observational evidence further suggests particular effectiveness for axial LBP when T12 or upper‐lumbar ganglia are targeted [13, 14, 15, 16, 17, 18, 19, 20, 21, 22, 23]. Nevertheless, the durability of benefit beyond 1 year and the real‐world safety profile remain poorly characterized. Prospective single‐centre cohorts across diverse indications—not limited to LBP—have reported sustained analgesia at 24 months [24]. Small mixed‐etiology series have followed responders for up to 3 years [25, 26] but limited sample sizes and methodological heterogeneity hamper generalization. A 2024 systematic review concluded that high‐quality longitudinal data for DRG‐S in chronic LBP are “urgently needed” [27].

In 2024 we published the first real‐world registry analysis of bilateral T12 DRG‐S for predominantly low‐back pain in 11 consecutive patients, demonstrating a 71% mean pain reduction and multidimensional improvements at 6 months [13]. Since that report, our institutional quality registry has expanded substantially; more patients have been implanted under a uniform multidisciplinary protocol, and routine digital follow‐up now extends to 24 months. This study provides a unique opportunity to assess the effectiveness, safety, and long‐term sustainability of DRG‐S for intractable LBP in a real‐world clinical setting. Beyond pain relief, it also evaluates secondary outcomes, including health‐related quality of life and longitudinal medication use, with particular attention to potential opioid‐sparing effects.

This study presents updated real‐world registry data on bilateral T12 (± additional level(s)) DRG‐S for chronic low back pain, with or without associated leg pain. The primary objective was to evaluate clinical outcomes—including pain intensity, functional status, psychological well‐being, patient satisfaction, and sleep quality—over a 24‐month follow‐up period in a larger cohort than previously reported. Secondary objectives were to determine responder rates, assess the durability of therapeutic effects at 12 and 24 months, and characterize therapy‐related adverse events and device revisions within the context of routine clinical practice. At last, the study aimed to quantify longitudinal changes in analgesic use, with particular emphasis on opioid‐sparing outcomes.

By providing robust, longer‐term, real‐world evidence, we aim to clarify the role of DRG‐S in the therapeutic algorithm for persistent spinal pain syndrome affecting the lower back and to inform patient selection, counseling, and future controlled trials.

## 2 Method

### 2.1 Patients

Thirty‐three consecutive patients (8 men, 25 women; mean age 59.8 ± 10.9 years) with chronic LBP were referred to our center for consideration of DRG‐S. After multidisciplinary evaluation, 20 patients met the eligibility criteria for trial stimulation. Eligible candidates presented with predominant low back pain, with or without associated leg pain, and had completed a structured multimodal pain management program. All participants had exhausted less invasive treatment options prior to referral. Furthermore, each patient underwent a spinal surgical evaluation to confirm that no additional surgical intervention was indicated.

Exclusion criteria comprised standard contraindications to neuromodulation, including: active systemic or local infection; uncontrolled coagulopathy or severe thrombocytopenia; anticoagulant therapy that could not be safely interrupted; pregnancy; severe immunosuppression or other conditions conferring unacceptable surgical or anesthetic risk; anatomical abnormalities preventing safe lead placement; known allergy or hypersensitivity to device components; non‐compatible cardiac pacemakers or implantable cardioverter–defibrillators; uncontrolled major psychiatric illness or active suicidal ideation; untreated substance use disorder; and cognitive impairment or other factors precluding effective device management and follow‐up.

### 2.2 Surgical Techniques and Programming

DRG implantation was performed in two stages with an intervening trial period and all procedures were carried out under local anesthesia.

#### 2.2.1 Stage 1—Lead Placement

Two to four SlimTip leads (Abbott Laboratories, Plano, TX, USA) were implanted using a contralateral approach through Tuohy needles under fluoroscopic guidance and directed to the dorsal root ganglia corresponding to each patient's pain topography. In all cases, at least two T12 leads were placed to provide coverage for axial back pain. In patients with concomitant leg pain, a third lead was positioned at S1 on the painful side, whereas those with bilateral leg pain received two S1 leads, one for each side. For S1 placement, a retrograde contralateral approach was used until 2022; after which an ipsilateral transforaminal approach was adopted. One patient with bilateral groin and scrotal pain in addition to low back pain received bilateral L1 leads in combination with bilateral T12 leads (Table 2).

Of the 20 patients, 3 were implanted with four leads and 6 with three leads, whereas the remainder received two leads. The average number of leads implanted was 2.7 per patient (Table 2). Adequate positioning was confirmed in all cases by intraoperative test stimulation.

#### 2.2.2 Stage 2—Implantable Pulse Generator (IPG) Insertion or Lead Explantation

After a 7‐day trial, patients with ≥ 50% reduction in mean numerical rating scale (NRS) pain intensity were classified as responders and received a permanent Proclaim DRG IPG (Abbott Laboratories) implanted in a subcutaneous pocket. Non‐responders had their leads removed.

#### 2.2.3 Programming

Permanent systems were programmed to deliver paraesthesia‐free, sub‐threshold stimulation. All programming and follow‐up adjustments were conducted by two specialist neuromodulation nurses with more than 5 years of experience in DRG therapy. At the last recorded programming session, 16 of 18 patients were receiving stimulation at 4 Hz, whereas only 2 patients were programmed at 20 Hz. The average pulse width across the cohort was 258 μs, with a mean amplitude of 0.29 mA.

### 2.3 Data Collection

At the Neurosurgical Department, Northern University Hospital (Sweden), all patients undergoing neuromodulation are monitored via the digital patient‐management platform Patientkollen (marketed outside Sweden as Caremetrix). Data generated through routine follow‐up are collected and stored in a dedicated local quality database, Neuropain.

All data for this study were prospectively collected using the digital platform Patientkollen/Caremetrix. The system automatically notified patients when it was time to complete questionnaires at predefined data collection time points. Patients accessed the platform through secure login and submitted their responses electronically. All data were securely stored in a local quality registry. This digital approach facilitated a high questionnaire completion rate (Table 1).

**TABLE 1:** Questionnaire completion over time.

Time point	Subjects expected	Subjects completed	Completion rate (%)
Baseline	20	20	100
3 months	18	18	100
6 months	18	18	100
12 months	16	16	100
24 months	13	10	77

*Note:* Completion rates of patient‐reported outcome measures were high throughout follow‐up, with 100% response at baseline, 3, 6, and 12 months. At 24 months, questionnaires were completed by 10 of 13 eligible patients, corresponding to a completion rate of 77%.

#### 2.3.1 Trial Period

During the 7‐day trial, participants completed a daily electronic diary capturing pain intensity, mood, activities of daily living (ADL), social participation, and sleep quality. Improvements in any of these domains were recorded as supporting evidence of a positive response.

Eighteen of the 20 patients met the responder criterion (≥ 50% reduction in mean NRS pain intensity) and also showed clinically meaningful gains in mood, ADL, social participation, and sleep. These 18 patients proceeded to a permanent implant.

#### 2.3.2 Follow Up

##### 2.3.2.1 Efficacy Data and Satisfaction

All patients were followed up at 3, 6, 12, and 24 months via digital questionnaires. Outcomes were compared with baseline values collected 4–6 days before implantation. The primary outcome was pain intensity on an 11‐point NRS: 0–10.

Secondary outcomes included pain catastrophising measured by the Pain Catastrophizing Scale (PCS) and health‐related quality of life assessed with the Patient‐Reported Outcomes Measurement Information System 29, version 2.1 (PROMIS‐29 v2.1). In addition, patients rated overall satisfaction with stimulation therapy on a 0–10 NRS.

##### 2.3.2.2 Analgesic Medication Use

Medication use was recorded at baseline, 1‐, and 2‐year follow‐up visits using patient‐reported data. All reported medications were reviewed and classified into predefined drug classes: opioids, gabapentinoids, paracetamol, antidepressants, muscle relaxants, COX‐2 inhibitors/non‐steroidal anti‐inflammatory drugs (NSAIDs), and hypnotics. For opioids, daily doses were converted into oral morphine equivalent doses (MED, mg/day) using standard conversion factors. For other drug classes, daily doses were recorded in milligrams.

If a patient had available medication data for a given time point but was not prescribed a drug within a class, the daily dose was recorded as zero. Data points were excluded if information on dose or regimen was missing or unclear. Cases with no medication data available for a given time point were excluded from analyses for that time point.

##### 2.3.2.3 Adverse Events and End‐of‐Treatment

During follow‐up all adverse events (AEs) and end‐of‐treatment (EOT) events were recorded in real time using the *Patientkollen*/*Caremetrix platform*. For each entry, the following fields were captured: (i) date of occurrence; (ii) AE type/description; (iii) implant components involved (e.g., lead, extension, implantable pulse generator [IPG], pocket); (iv) whether the event appeared related to any specific factor or precipitating circumstance (e.g., procedure, infection, trauma, device handling); (v) management (e.g., observation, medication, reprogramming, surgical revision, explantation); and (vi) outcome (resolved/ongoing/EOT). Definitions were prespecified prior to analysis: an AE was any untoward medical occurrence after implantation irrespective of causality, and EOT was defined as permanent discontinuation of stimulation for any clinical reason.

In this cohort, seven complications were registered in Patientkollen and are presented in Table 2, listing for each case when the AE happened, AE type, involved component(s), relatedness, management, and outcome/EOT status. Two participants met EOT criteria: one due to progressive olisthesis requiring spine surgery (unrelated to the neuromodulation system), and one due to a rare cutaneous lymphoma with skin erosion over the IPG, necessitating explantation (Table 3).

**TABLE 2:** Patient demographics and clinical characteristics.

Parameter	Value
Mean age (±SD)	59.8 (±10.9)
Gender	10 female/8 men
Indication	3 PSPS 1/15 PSPS 2
Isolated back pain	7
Back pain + groin/leg pain	11
Mean number of implanted leads	2.7

*Note:* This table provides an overview of the demographic and clinical characteristics of the patient cohort. Data includes age, gender distribution, clinical indications for treatment, pain localization, and the mean number of implanted leads.

**TABLE 3:** (a) Adverse events. (b) End‐of‐treatment (EOT).

(a)							
AE ID	Timing	Type of AE	Involved component(s)/factor(s)	External circumstance	Management	Outcome for therapy	Time from implant to complication (days)
1	Later than 3 months post‐implant	Lead migration	Lead(s), external circumstance	Slip accident	Planned revision with component/system replacement to restore therapy	Therapy restored without sequelae	161
2	Later than 3 months post‐implant	Lead migration	Lead(s), external circumstance	Fall accident, slipped on ice	Planned revision with component/system replacement to restore therapy	Therapy restored without sequelae	306
3	Later than 3 months post‐implant	Lead migration	Lead(s), external circumstance	Hospitalization; lead migrated during transfer between beds	Planned revision with component/system replacement to restore therapy	Therapy restored without sequelae	146
4	During in‐hospital period (incl. trial)	Lead fracture/damage—high impedance	Lead(s), external circumstance	Fall	Planned revision with component/system replacement to restore therapy	Therapy restored without sequelae	121
5	Later than 3 months post‐implant	Lead fracture/damage—high impedance	Lead(s), external circumstance	Fall	Planned revision with component/system replacement to restore therapy	Therapy restored without sequelae	638
6	Later than 3 months post‐implant	Lead fracture/damage—high impedance	Lead(s)	Not specified	Planned revision with component/system replacement to restore therapy	Therapy restored without sequelae	231
7	Later than 3 months post‐implant	Lead fracture/damage—high impedance	Lead(s), external circumstance	Not specified	Planned revision with component/system replacement to restore therapy	Therapy restored without sequelae	952

(b)		
EOT ID	Time from implant to EOT (days)	Reason for EOT
1	847	Progressive spondylolisthesis; need for spinal surgery
2	770	Skin lymphoma with erosion over IPG; system explanted

*Note:* (a) summarizes all adverse events (AEs) captured prospectively in the Patientkollen system. For each AE we list timing relative to implantation, the complication type, involved component(s) or factors, any external precipitating circumstance, clinical management, and the outcome in relation to stimulation therapy. (b) lists patients who reached end‐of‐treatment (EOT), defined as permanent discontinuation of stimulation. Time from implantation to EOT is presented, and the clinical reason for EOT.

In addition to descriptive reporting, AE data will be evaluated using Kaplan–Meier methods to characterize time‐to‐event (e.g., time to first complication) and complication‐free survival.

### 2.4 Statistical Analysis

Descriptive statistics were used to summarize baseline demographic and clinical characteristics, presented as means with standard deviations or as medians with ranges for continuous variables. Changes in patient‐reported outcome measures over time were evaluated using paired *t*‐tests, comparing each follow‐up time point (90, 180, 365, and 730 days) with baseline values. This approach was applied to all PROMIS‐29 domains as well as to the PCS total and subscale scores. Statistical significance was defined as a two‐sided *p*‐value of < 0.05. Time to complication was analyzed using Kaplan–Meier survival analysis. All statistical analyses were conducted using SPSS, version 29.0.2.0 (IBM Corp).

Medication data were analyzed separately. For each drug class and time point, the number of patients with available data (*n*), mean daily dose, median daily dose, and range (minimum–maximum) were calculated. Within‐patient changes from baseline to the 1‐ and 2‐year follow‐ups were assessed using Wilcoxon signed‐rank tests, applying a two‐sided significance threshold of *p* < 0.05.

### 2.5 Ethical Considerations

Data extraction from the Neuropain quality database was conducted under approval from the Swedish Ethical Review Authority (Dnr 2022‐05623‐01) and in accordance with the Declaration of Helsinki, EU GDPR, and applicable Swedish regulations. Individual patient consent was not required because the study used data from a quality registry with established legal grounds for processing. Prior to registry inclusion, all patients receive information about data collection, purposes, rights (including access, rectification, and objection), and data protection. Data handling followed principles of purpose limitation, data minimization, and secure storage.

## 3 Results

Of the 20 patients who underwent trial stimulation, 18 (90%) achieved a successful trial and subsequently received permanent IPG.

### 3.1 Pain Intensity (NRS)

At baseline, the mean Numeric Rating Scale (NRS) score for pain was 8.0 ± 1.8 (*n* = 20), with a median of 8.0 (IQR 6.5–9.5) and a range of 4–10. Substantial and statistically significant reductions in pain were observed across all follow‐up assessments (*p* < 0.001 for all comparisons vs. baseline) (Table 4).

**TABLE 4:** Numeric Rating Scale (NRS) scores for pain intensity at baseline and follow‐up.

NRS summary for subjects	Baseline	3‐months	6‐months	12‐months	24‐months
Numeric Rating Scale					
Mean ± SD (*n*)	8 ± 1.8 (20)	3.6 ± 1.6 (18)	3.2 ± 1.8 (18)	3.6 ± 1.7 (16)	2.9 ± 1.6 (10)
Median	8 (6.5, 9.5)	3.5 (2, 4)	3 (1, 4)	4 (2, 5)	2.5 (2, 4)
Range (min, max)	(4, 10)	(1, 8)	(1, 6)	(1, 6)	(1, 6)
Numeric Rating Scale Δ (%)					
Mean ± SD (*n*)	(0)	55.1 ± 22 (18)	60.8 ± 23.6 (18)	55.2 ± 21 (16)	65 ± 19.7 (10)
Median		57.8 (50, 71.4)	64.6 (50, 80)	55 (38.7, 72.2)	68.8 (44.4, 80)
Range (min, max)		(0, 80)	(0, 90)	(16.7, 90)	(33.3, 90)
*p*		< 0.01	< 0.01	< 0.01	< 0.01
Numeric Rating Scale Δ (%) 30%		15 (83.3%)	15 (83.3%)	14 (87.5%)	10 (100%)
Numeric Rating Scale Δ (%) 50%		14 (77.8%)	15 (83.3%)	10 (62.5%)	7 (70%)

*Note:* The table presents mean, median, and range of NRS pain scores at baseline, 3, 6, 12, and 24 months. It also includes the number of patients with available data at each time point, as well as the proportions of patients achieving ≥ 30% and ≥ 50% pain reduction compared with baseline.

At 3 months, the mean NRS score decreased to 3.6 ± 1.6 (*n* = 18), corresponding to a mean relative reduction of 55.1% from baseline (*p* < 0.001). At 6 months, the mean was 3.2 ± 1.8 (*n* = 18; 60.8% reduction, *p* < 0.001), and at 12 months, 3.6 ± 1.7 (*n* = 16; 55.2% reduction, *p* < 0.001). By 24 months, the mean NRS score was 2.9 ± 1.6 (*n* = 10), representing a 65.0% reduction (*p* < 0.001). Median scores followed a similar pattern, declining to 3.5 at 3 months, 3.0 at 6 months, 4.0 at 12 months, and 2.5 at 24 months, with observed ranges narrowing to 1–6 at follow‐ups.

Responder analysis demonstrated that a large proportion of patients achieved clinically meaningful pain relief. At least 30% reduction in NRS was reported in 83.3% (15/18) of patients at both 3 and 6 months, 87.5% (14/16) at 12 months, and 100% (10/10) at 24 months. A 50% or greater reduction was observed in 77.8% (14/18) at 3 months, 83.3% (15/18) at 6 months, 62.5% (10/16) at 12 months, and 70% (7/10) at 24 months.

Together, these results indicate a robust and durable reduction in pain intensity over 2 years, with the majority of patients experiencing clinically meaningful improvement.

### 3.2 PROMIS‐29

Patient‐reported outcomes demonstrated broad and sustained improvements across multiple PROMIS‐29 domains over the 24‐month follow‐up.

Physical function improved from a baseline mean t‐score of 35.1–42.0 at 24 months, with statistically significant gains evident as early as 3 months (Δ +5.7; *p* < 0.001) and maintained thereafter.

Pain interference scores declined from 69.1 at baseline to 58.7 at both 12 (*p* < 0.001) and 24 months (all *p* = 0.004), reflecting reduced disruption of daily life. Pain intensity likewise decreased from 8.2 at baseline to 4.3 at 12 months (*p* < 0.001) and 3.9 at 24 months (*p* < 0.001).

Improvements were also seen in psychosocial domains. Anxiety declined from 60.7 to 52.8 at 24 months, with significant reductions at interim time points up to 12 months (*p* ≤ 0.006). Depression decreased from 58.7 to 54.6, with significant improvements through 12 months (*p* ≤ 0.006), though the 24‐month difference did not reach significance.

Fatigue improved from 60.3 at baseline to 51.9 at 24 months, with significant improvements observed at all follow‐up points (*p* ≤ 0.01). Sleep disturbance scores decreased from 60.0 to 51.7 over the same period, with significant improvements evident at each follow‐up (*p* ≤ 0.01).

Finally, the ability to participate in social roles and activities increased from a mean of 37.7 to 45.0, with progressive and statistically significant gains across follow‐up (*p* ≤ 0.001).

Taken together, these PROMIS‐29 results highlight consistent and clinically meaningful improvements across physical, emotional, and social domains, with the most robust effects observed for physical function, pain interference, fatigue, and sleep disturbance (Table 5).

**TABLE 5:** PROMIS‐29 domain outcomes at baseline and follow‐up (3, 6, 12, and 24 months).

Promis‐29 domains	Baseline	3 months	6 months	1 year	2 years
Physical function					
Mean ± SD (*n*)	35.1 ± 5.5 (20)	40.1 ± 5 (18)	42.5 ± 5.9 (18)	40.4 ± 4.8 (16)	42 ± 7.7 (10)
Median	35 (30.5, 40.5)	40.5 (35.6, 41.9)	40.5 (39.2, 48.3)	39.9 (36.7, 44.5)	42.7 (36.7, 45.5)
Range (min, max)	(26.6, 43.5)	(30.5, 48.3)	(31.9, 57)	(31.9, 48.3)	(30.5, 57)
Physical function Δ					
Mean ± SD (*n*)		5.7 ± 4.9 (18)	8.1 ± 5.8 (18)	6.5 ± 5.2 (16)	9.4 ± 7.9 (10)
Median		3.9 (1.4, 7.8)	6.3 (4.8, 13.9)	6.1 (3.1, 7.8)	6.2 (3.6, 15.3)
Range (min, max)		(−1.6, 16.4)	(−2.5, 16.6)	(−1.2, 19.4)	(1.6, 28.1)
*p*		< 0.001	< 0.001	< 0.001	0.006
Anxiety					
Mean ± SD (*n*)	60.7 ± 9.1 (20)	52.9 ± 8.9 (18)	50.7 ± 8.9 (18)	51 ± 9.1 (16)	52.8 ± 11.3 (10)
Median	61.4 (55.7, 65.35)	55.8 (40.3, 59.5)	51.2 (40.3, 57.7)	48 (40.3, 59.5)	48 (40.3, 65.3)
Range (min, max)	(40.3, 75.4)	(40.3, 67.3)	(40.3, 69.3)	(40.3, 67.3)	(40.3, 71.2)
Anxiety Δ					
Mean ± SD (*n*)		−8.2 ± 10.4 (18)	−10.4 ± 10.6 (18)	−9.8 ± 11.8 (16)	−9.2 ± 14.7 (10)
Median		−5.8 (−15.4, −1.8)	−7.7 (−17.7, 0)	−7.7 (−20.15, 0)	−7 (−19.2, 0)
Range (min, max)		(−35.1, 7.8)	(−35.1, 3.2)	(−33, 11.5)	(−33, 17.3)
*p*		0.005	< 0.001	0.006	0.095
Depression					
Mean ± SD (*n*)	58.7 ± 9.6 (20)	52.8 ± 8.4 (18)	52 ± 8.8 (18)	52 ± 9 (16)	54.6 ± 10.7 (10)
Median	58.1 (52.85, 65.7)	55.7 (41, 58.9)	54.8 (41, 60.5)	50.4 (41, 60.55)	53.8 (41, 65.7)
Range (min, max)	(41, 79.4)	(41, 65.7)	(41, 65.7)	(41, 65.7)	(41, 69.4)
Depression Δ					
Mean ± SD (*n*)		−6.2 ± 7.6 (18)	−7 ± 8.6 (18)	−7 ± 8.4 (16)	−4.9 ± 10.9 (10)
Median		−5.9 (−10.8, 0)	−5.9 (−14.7, 0)	−9.3 (−13.45, −0.9)	−7 (−13.7, 0)
Range (min, max)		(−22.2, 9.9)	(−22.2, 6.7)	(−17.6, 14.9)	(−19.5, 20.4)
*p*		0.004	0.004	0.006	0.21
Fatigue					
Mean ± SD (*n*)	60.3 ± 9.8 (20)	53.5 ± 8 (18)	52.1 ± 10.4 (18)	51.5 ± 11.3 (16)	51.9 ± 10.4 (10)
Median	64.6 (52.05, 65.65)	49.8 (48.6, 62.7)	55.1 (46, 60.7)	50.9 (43.1, 58.85)	48.6 (43.1, 64.6)
Range (min, max)	(39.7, 75.8)	(43.1, 69)	(33.7, 69)	(33.7, 75.8)	(39.7, 64.6)
Fatigue Δ					
Mean ± SD (*n*)		−8 ± 5.9 (18)	−9.4 ± 10.7 (18)	−11.1 ± 9.1 (16)	−9.9 ± 7.8 (10)
Median		−7.1 (−13.9, −2.9)	−7.6 (−14.9, −2)	−6.7 (−17.7, −3.95)	−10.6 (−16, −2.9)
Range (min, max)		(−18, 2.3)	(−30.9, 9.5)	(−30.9, 0)	(−24.9, 1.9)
*p*		< 0.001	0.002	< 0.001	0.01
Sleep disturbance					
Mean ± SD (*n*)	60 ± 8.8 (20)	54.6 ± 6.9 (18)	53.5 ± 5.7 (18)	53.1 ± 5 (16)	51.7 ± 5.7 (10)
Median	61.7 (56.1, 66)	55.2 (48.4, 59.8)	54.3 (46.2, 57.9)	53.4 (50.4, 57.9)	53.4 (46.2, 56.1)
Range (min, max)	(41.1, 73.3)	(43.8, 63.8)	(43.8, 61.7)	(43.8, 59.8)	(41.1, 59.8)
Sleep disturbance Δ					
Mean ± SD (*n*)		−6 ± 5.7 (18)	−7 ± 7 (18)	−8.6 ± 7.1 (16)	−9.1 ± 7.6 (10)
Median		−3.9 (−9.9, −2.4)	−7.9 (−11.6, −1.8)	−11.6 (−13.85, −3.8)	−9.6 (−11.7, −5.6)
Range (min, max)		(−16.4, 7.3)	(−19.8, 6.2)	(−17.2, 5.1)	(−25, 5.1)
*p*		< 0.001	0.011	0.04	0.27
Ability to participate in social roles and activities					
Mean ± SD (*n*)	37.7 ± 5.1 (20)	43.2 ± 5.8 (18)	45.5 ± 7 (18)	43.9 ± 5.9 (16)	45 ± 5.5 (10)
Median	37.3 (34.85, 41.4)	44.2 (38.8, 46.2)	44.2 (42.3, 50)	44.2 (40.5, 47.15)	47.2 (38.8, 50)
Range (min, max)	(27.5, 46.2)	(27.5, 51.9)	(34, 64.2)	(34, 55.8)	(35.7, 51.9)
Ability to participate in social roles and activities Δ					
Mean ± SD (*n*)		6.2 ± 4.9 (18)	8.6 ± 4.8 (18)	7.4 ± 5.9 (16)	9 ± 5.8 (10)
Median		6.8 (2, 10.2)	9 (5, 10.5)	7.9 (3.7, 11.6)	9.8 (3.7, 12.4)
Range (min, max)		(−3.5, 14.1)	(0, 18.5)	(−3.3, 18)	(0, 18.2)
*p*		< 0.001	< 0.001	< 0.001	< 0.001
Pain interference					
Mean ± SD (*n*)	69.1 ± 5.5 (20)	61.7 ± 6.3 (18)	58.3 ± 6.7 (18)	58.7 ± 7.1 (16)	58.7 ± 4.2 (10)
Median	69.7 (65.2, 75.6)	61.2 (59.9, 65.2)	58.5 (57.1, 61.2)	57.1 (55.6, 63.8)	57.8 (57.1, 62.5)
Range (min, max)	(58.5, 75.6)	(41.6, 71.6)	(41.6, 68)	(41.6, 75.6)	(49.6, 65.2)
Pain interference Δ					
Mean ± SD (*n*)		−8.4 ± 5.2 (18)	−11.8 ± 5.8 (18)	−11.8 ± 5.5 (16)	−12.5 ± 5.6 (10)
Median		−7.7 (−11.7, −6.7)	−10.8 (−17.1, −6.9)	−11.8 (−15.7, −9.1)	−11.5 (−18.5, −8.1)
Range (min, max)		(−17.1, 1.9)	(−23.6, −2.8)	(−20, 0)	(−22, −4)
*p*		0.031	0.002	< 0.001	0.004
Pain intensity					
Mean ± SD (*n*)	8 ± 1.7 (20)	4.2 ± 2.1 (18)	3.7 ± 1.8 (18)	4.3 ± 1.6 (16)	3.9 ± 1.1 (10)
Median	9 (7, 9)	4 (3, 5)	4 (2, 5)	4.5 (3, 5.5)	4 (3, 5)
Range (min, max)	(4, 10)	(1, 8)	(1, 7)	(2, 7)	(2, 6)
Pain intensity Δ					
Mean ± SD (*n*)		−4.1 ± 2.5 (18)	−4.5 ± 2.5 (18)	−4.1 ± 2 (16)	−4.8 ± 1.8 (10)
Median		−5 (−5, −2)	−4 (−6, −4)	−3.5 (−6, −2.5)	−5 (−7, −4)
Range (min, max)		(−9, 0)	(−9, 1)	(−8, −1)	(−7, −1)
*p*		< 0.001	< 0.001	< 0.001	< 0.001

*Note:* Values are presented as mean ± SD (*n*), median (IQR), and range (min–max). Δ values represent the change from baseline. Clinically meaningful improvements were seen across multiple domains, with the largest changes in physical function, pain interference, and pain intensity.

### 3.3 Pain Catastrophizing Scale

At baseline, patients reported high levels of pain catastrophizing across all PCS domains, with a total mean score of 25.6 (SD 11.1). Substantial and statistically significant reductions were observed over time. By 3 months, the PCS total score had decreased to a mean of 14.6, and this improvement was sustained at 6, 12, and 24 months (means 11.1, 11.0, and 11.8, respectively; all *p* < 0.001 vs. baseline). Median values showed similar reductions, from 27 at baseline to 11.5 at 24 months (Table 6).

**TABLE 6:** Pain Catastrophizing Scale (PCS) outcomes at baseline and follow‐up (3, 6, 12, and 24 months).

PCS	Baseline	3 months	6 months	1 year	2 years
PCS total					
Mean ± SD (*n*)	25.6 ± 11.1 (20)	14.6 ± 9.9 (18)	11.1 ± 8.6 (18)	11 ± 8.4 (16)	11.8 ± 8.4 (10)
Median	27 (16.5, 32.5)	14 (7, 23)	10 (3, 17)	9 (4.5, 17.5)	11.5 (5, 18)
Range (min, max)	(8, 47)	(1, 34)	(0, 27)	(0, 28)	(0, 27)
PCS total Δ %					
Mean ± SD (*n*)	(0)	47.9 ± 30.4 (18)	62 ± 25.3 (18)	56.9 ± 30.9 (16)	59.6 ± 28.5 (10)
Median	()	50.5 (36.36, 70.97)	62.2 (44.44, 76.92)	53.5 (32.29, 83.24)	60.2 (44.44, 74.19)
Range (min, max)		(−15, 88.9)	(13.8, 100)	(3.5, 100)	(10, 100)
*p*		< 0.001	< 0.001	< 0.001	< 0.001
Helplessness					
Mean ± SD (*n*)	12.7 ± 5.6 (20)	6.5 ± 4.9 (18)	5.1 ± 4 (18)	5.4 ± 3.8 (16)	6.2 ± 4.6 (10)
Median	13.5 (7, 16.5)	6 (1, 10)	5.5 (1, 8)	5 (3, 9)	5 (4, 10)
Range (min, max)	(1, 23)	(0, 16)	(0, 12)	(0, 12)	(0, 14)
Helplessness Δ %					
Mean ± SD (*n*)	(0)	51.2 ± 31.6 (18)	65.5 ± 25.7 (18)	42.1 ± 70 (16)	50.9 ± 42.5 (10)
Median	()	52.7 (33.33, 75)	63.9 (52.17, 91.67)	57.6 (30.355, 82.29)	64.9 (16.67, 80.95)
Range (min, max)		(−6.7, 100)	(20, 100)	(−200, 100)	(−42.9, 100)
*p*		< 0.001	< 0.001	< 0.001	0.004
Magnification					
Mean ± SD (*n*)	4.3 ± 2.7 (20)	3 ± 2.4 (18)	1.9 ± 2.2 (18)	2.2 ± 2 (16)	1.7 ± 1.7 (10)
Median	3.5 (2.5, 6)	3 (1, 4)	1 (0, 3)	1.5 (0.5, 3.5)	1.5 (0, 3)
Range (min, max)	(0, 9)	(0, 9)	(0, 7)	(0, 7)	(0, 5)
Magnification Δ %					
Mean ± SD (*n*)	(0)	29.4 ± 47.5 (18)	52.7 ± 36.1 (18)	31.2 ± 80.3 (16)	64.8 ± 33 (10)
Median	()	33.3 (0, 66.67)	58.3 (22.22, 83.33)	38.8 (12.5, 81.745)	66.7 (50, 100)
Range (min, max)		(−50, 100)	(−16.7, 100)	(−250, 100)	(0, 100)
*p*		0.072	< 0.001	0.013	< 0.001
Rumination					
Mean ± SD (*n*)	8.6 ± 3.9 (20)	5.1 ± 3.9 (18)	4.1 ± 3.5 (18)	3.4 ± 3 (16)	3.9 ± 3 (10)
Median	8.5 (5, 12)	4.5 (2, 7)	3.5 (2, 6)	2 (1, 5.5)	4.5 (0, 6)
Range (min, max)	(2, 16)	(0, 15)	(0, 13)	(0, 10)	(0, 9)
Rumination Δ %					
Mean ± SD (*n*)	(0)	40.2 ± 45.9 (18)	56.2 ± 28.6 (18)	60 ± 38.3 (16)	61.8 ± 35.2 (10)
Median	()	37.5 (21.43, 75)	55.9 (33.33, 71.43)	66.7 (41.665, 90.91)	65.5 (45.45, 100)
Range (min, max)		(−75, 100)	(−8.3, 100)	(−50, 100)	(−16.7, 100)
*p*		0.002	< 0.001	< 0.001	0.002

*Note:* Values are presented as mean ± SD (*n*), median (IQR), and range (min–max). Δ values represent the percentage change from baseline. Statistically significant reductions were observed across the total PCS score and all subdomains (helplessness, magnification, and rumination), indicating a sustained decrease in maladaptive pain‐related cognitions.

Across individual domains, consistent improvements were also observed. Helplessness, the highest scoring subdomain at baseline (mean 12.7), showed a marked reduction to 6.5 at 3 months and remained stable thereafter (5.1–6.2 through 24 months, all *p* < 0.01). Magnification decreased from 4.3 at baseline to 1.7 at 24 months, with significant improvements already evident at 6 months (*p* < 0.001). Rumination followed a similar trend, with scores declining from 8.6 at baseline to 3.9 at 24 months (all *p* = 0.002).

The proportion of change (Δ values) further confirmed these improvements, with PCS total score decreased by 47%–62% across follow‐ups compared to baseline. The most pronounced relative improvements were observed in the helplessness and rumination domains, where median reductions exceeded 50% already by 6 months.

### 3.4 Patient Satisfaction

Patient‐reported satisfaction with treatment remained consistently high throughout follow‐up (Table 7). At 3 months, the mean satisfaction score was 8.0 ± 1.8 (*n* = 15), with a median of 8 (range: 4–10). These values were stable at subsequent assessments: 7.8 ± 2.1 at 6 months (*n* = 18), 7.9 ± 1.7 at 12 months (*n* = 16), 8.2 ± 1.9 at 24 months (*n* = 10), and 8.0 ± 2.4 at 36 months (*n* = 6). Median scores were consistently ≥ 8 across all time points, and the range remained wide [3, 4, 5, 6, 7, 8, 9, 10], reflecting inter‐individual variability.

**TABLE 7:** General satisfaction with the therapy at follow‐up assessments.

Measure	3 months	6 months	12 months	24 months	36 months
Mean ± SD (*n*)	8 ± 1.8 (15)	7.8 ± 2.1 (18)	7.9 ± 1.7 (16)	8.2 ± 1.9 (10)	8 ± 2.4 (6)
Median	8 (6, 10)	8 (6, 10)	8 (7, 9.5)	8.5 (7, 10)	8.5 (8, 10)
Range (min, max)	(4, 10)	(4, 10)	(4, 10)	(4, 10)	(3, 10)

*Note:* The table presents mean ± SD (*n*), median (IQR), and range (minimum–maximum) satisfaction scores at 3, 6, 12, 24, and 36 months.

### 3.5 Analgesic Medication Use

Medication data were available for 13 patients at baseline, 12 at the 1 year, and 9 at the 2‐year follow‐up (Table 8).

**TABLE 8:** Overview table—Selected drug classes (corrected *p*‐values).

Drug class	Baseline	1 year	2 years
Opioids (MED mg/day)	*n* = 13	*n* = 12	*n* = 9
	Mean: 27.0	Mean: 11.25	Mean: 4.44
	Median: 30.0	Median: 12.5	Median: 0.0
	Range: 0.0–60.0	Range: 0.0–30.0	Range: 0.0–20.0
		*p* = 0.019	*p* = 0.011
Gabapentinoids (mg/day)	*n* = 13	*n* = 12	*n* = 9
	Mean: 515.38	Mean: 200.0	Mean: 233.33
	Median: 0.0	Median: 0.0	Median: 0.0
	Range: 0.0–2400.0	Range: 0.0–900.0	Range: 0.0–900.0
		*p* = 0.112	*p* = 0.141
Paracetamol (mg/day)	*n* = 13	*n* = 12	*n* = 9
	Mean: 2769.23	Mean: 1000.0	Mean: 1111.11
	Median: 4000.0	Median: 0.0	Median: 0.0
	Range: 0.0–8000.0	Range: 0.0–4000.0	Range: 0.0–4000.0
		*p* = 0.046	*p* = 0.026

*Note:* This table presents the daily consumption of three major drug classes (opioids, gabapentinoids, and paracetamol) at baseline, 1‐, and 2‐year follow‐ups. For each time point, the number of patients with available data (*n*), mean dose, median dose, and range are shown. *p*‐values were calculated using Wilcoxon signed‐rank tests comparing baseline values with those at 1‐ and 2‐year follow‐up.

**FIGURE 1:**
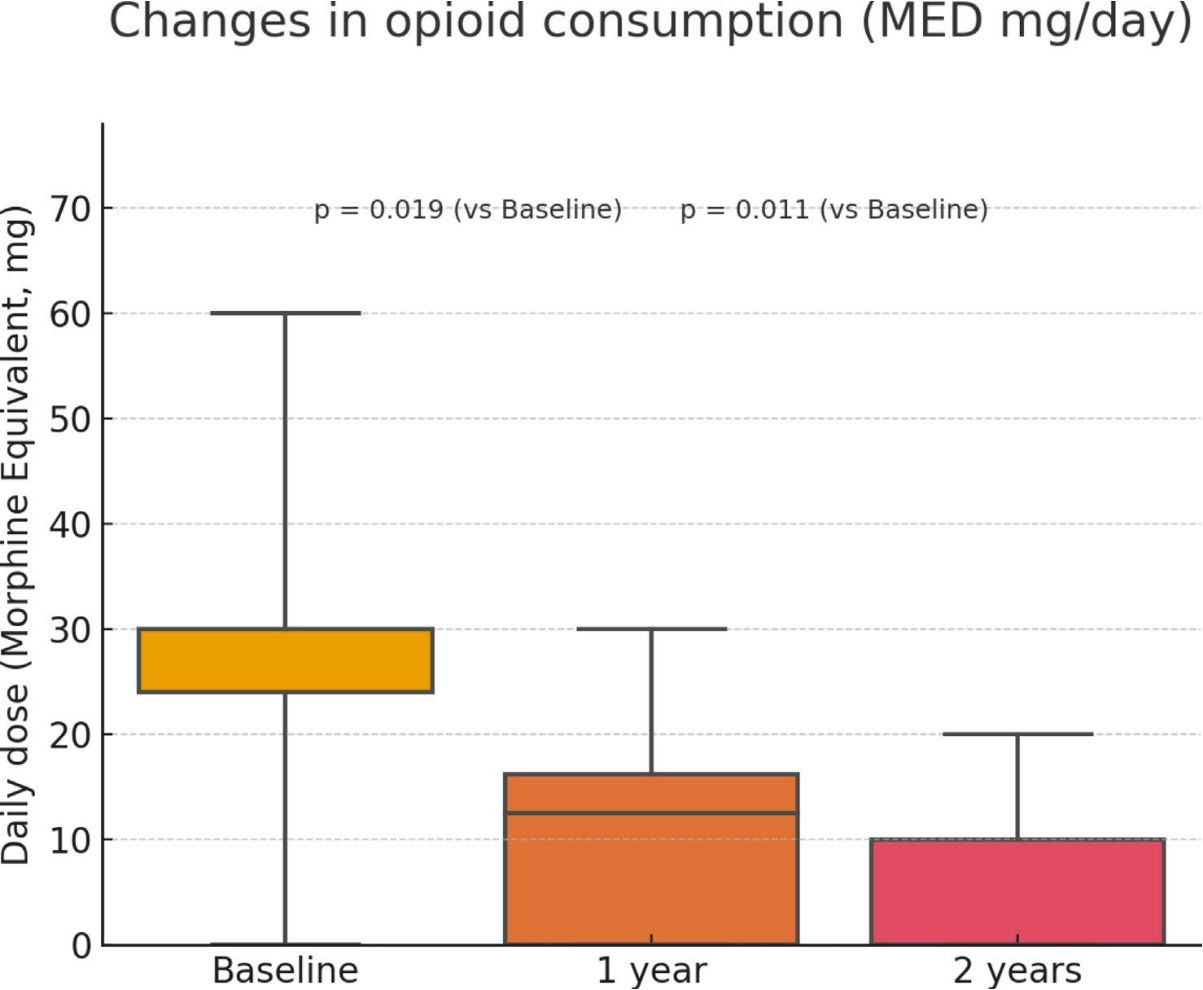
Changes in opioid consumption over time. Distribution of daily morphine equivalent dose (MED, mg/day) at baseline, 1‐, and 2‐year follow‐ups. All individual data points are shown; whiskers indicate the full range (min–max). Opioid use was significantly reduced at 1 year (*p* = 0.019) and 2 years (*p* = 0.011) compared with baseline.

Opioid consumption, expressed as daily morphine equivalent dose (MED, mg/day), showed a marked decline over time with mean dose falling from 27.0 mg at baseline to 11.25 mg at 1 year and 4.44 mg at 2 years. Median values followed a similar pattern, falling from 30.0 mg/day at baseline to 12.5 mg/day at 1 year and 0 mg/day at 2 years. These reductions were statistically significant at both follow‐up time points (1 year: *p* = 0.019; 2 years: *p* = 0.011) (Figure 1).

Gabapentinoid use varied substantially between individuals and did not show significant changes over time. The mean daily dose decreased from 515.38 mg at baseline to 200.0 mg at 1 year and 233.33 mg at 2 years, whereas the median remained 0.0 mg at all time points (*p* = 0.112 and *p* = 0.141 for 1 and 2 years vs. baseline, respectively) (Table 8).

Paracetamol use also decreased over time, with mean daily doses declining from 2769.23 mg at baseline to 1000.0 mg at 1 year and 1111.11 mg at 2 years. The median fell from 4000.0 mg at baseline to 0.0 mg at both follow‐ups. These reductions were statistically significant at both 1 year (*p* = 0.046) and 2 years (*p* = 0.026) (Table 8).

Other medication classes, including antidepressants, muscle relaxants, COX‐2 inhibitors/NSAIDs, and hypnotics, showed no statistically significant changes in daily dose between baseline and follow‐up assessments. Use within these categories remained low or absent in most patients throughout the study period.

### 3.6 Adverse Events and End‐of‐Treatment

A total of seven adverse events (AEs) were recorded during the study period, all of which were hardware‐related complications involving either lead migration (*n* = 3) or lead fracture/damage presenting as high impedances (*n* = 4). The complications occurred at variable times after implantation, ranging from 121 to 952 days post‐implant. In several cases, an external precipitating factor was identified, such as falls, slips, or hospital transfers. All events were managed with planned revision surgery, typically involving replacement of the affected lead or system components. Importantly, therapeutic stimulation could be restored in every case, and no patient experienced permanent loss of therapy as a direct result of the complication (Table 3a).

**FIGURE 2:**
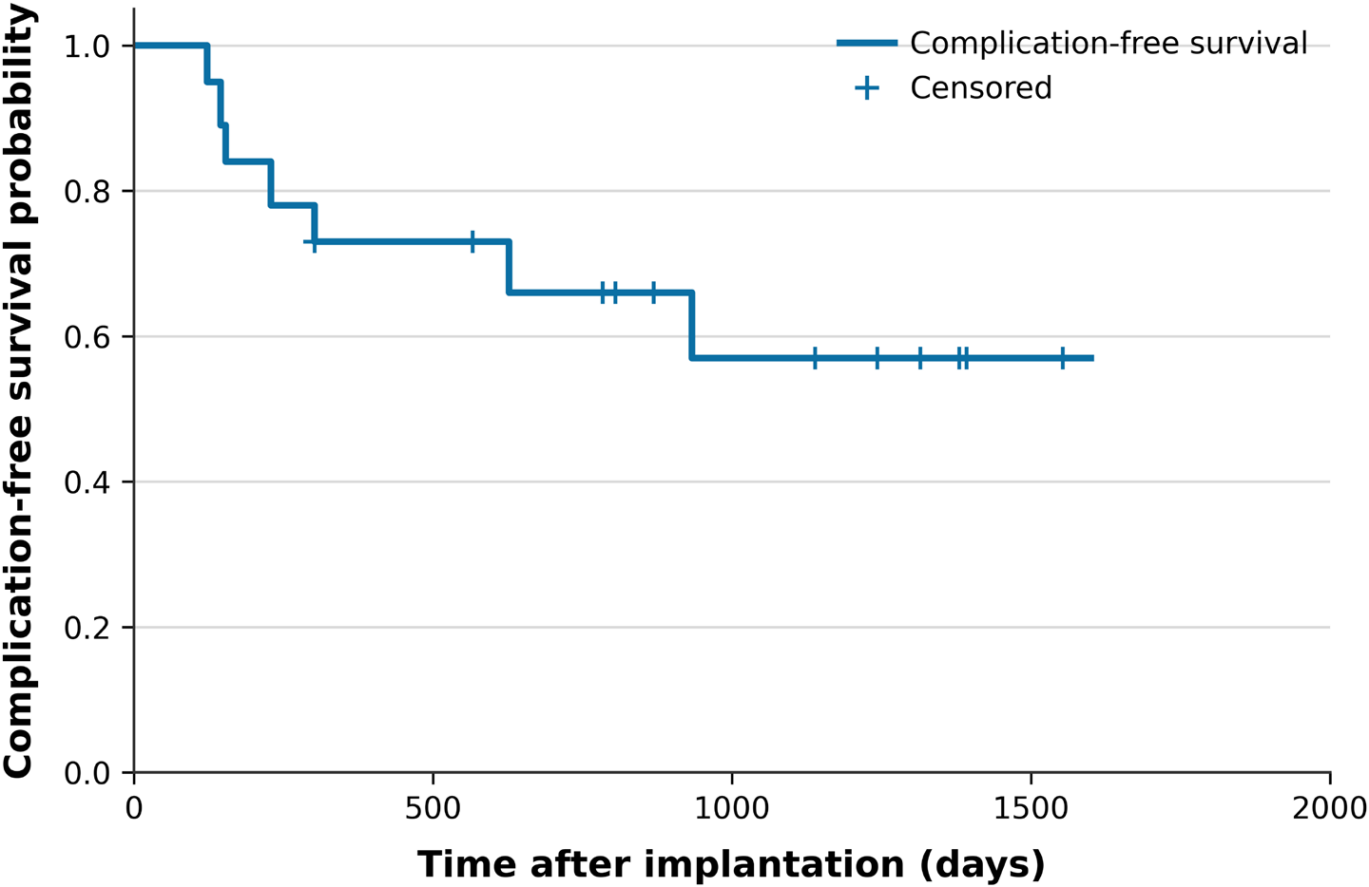
Kaplan–Meier analysis of time to complication after implantation. Seven complications were observed during follow‐up. The mean estimated time to complication was 1080 days (95% CI: 785–1375 days).

Kaplan–Meier survival analysis estimated the mean time to complication at 1080 days (95% CI: 785–1375 days) (Figure 2). This curve illustrates that although complications were not uncommon over longer follow‐up, they were consistently manageable with revision procedures.

Two patients reached end‐of‐treatment (EOT), defined as permanent discontinuation of stimulation. The first case was due to progressive spondylolisthesis necessitating spinal surgery (847 days after implantation). The second patient developed a rare cutaneous lymphoma with subsequent skin erosion over the IPG, leading to mandatory system explantation (770 days after implantation) (Table 3b). These patients were included in outcome analyses up to the time of treatment cessation and censored thereafter.

## 4 Discussion

This expanded prospective registry analysis of bilateral T12 DRG‐S for PSPS adds to the growing body of evidence supporting its long‐term effectiveness and safety. We observed significant and sustained improvements in pain intensity, function, psychological health, and quality of life over 2 years, accompanied by a meaningful reduction in opioid and paracetamol use. Importantly, these benefits were durable despite the modest sample size and the real‐world study context.

The observed reductions in pain intensity were consistent with earlier short‐term reports and align with previously published cohort studies, which suggested that DRG‐S at the T12 level may be particularly well‐suited for patients with axial LBP [13, 14, 17, 18]. Improvements extended beyond nociceptive outcomes to physical function, fatigue, sleep disturbance, and psychosocial well‐being, as reflected in the PROMIS‐29 and PCS assessments. This multidimensional response reinforces the notion that DRG‐S can positively influence domains of health beyond pain alone. Emerging mechanistic studies suggest that DRG‐S may modulate central sensitization, engage endogenous inhibitory pathways, and attenuate maladaptive pain cognitions, providing a neurophysiological basis for its broad therapeutic impact [28, 29].

In addition to improvements in pain, function, and psychological outcomes, patient satisfaction remained consistently high throughout follow‐up, with median ratings ≥ 8 across all time points up to 3 years. These findings underscore that the clinical benefits of DRG‐S translate into meaningful real‐world acceptance and patient‐perceived value of therapy, an important consideration for long‐term adherence and health economic evaluations.

One of the most clinically meaningful findings was the substantial reduction in opioid use, with mean MED decreasing from 27.0 mg/day at baseline to 4.44 mg/day at 24 months. This observation is of particular importance given the well‐documented risks associated with chronic opioid therapy including tolerance, dependence, and systemic adverse effects. The parallel reduction in opioid use with improvement in pain control and function suggests that DRG‐S may facilitate meaningful opioid‐sparing effects.

Although gabapentinoid use remained variable and non‐significant, and paracetamol consumption decreased modestly, the most robust signal of medication de‐escalation was observed with opioids. Our findings are consistent with prior studies reporting medication reduction following DRG‐S. Kretzschmar et al. [25] demonstrated a progressive decline in analgesic use, with most patients becoming opioid‐free by 36 months of follow‐up. Similarly, Graca et al. [30] found a marked reduction in morphine milligram equivalents among patients with CRPS following cervical and high‐thoracic DRG‐S. Taken together, these studies reinforce the observation that DRG‐S can provide durable analgesia while enabling clinically relevant reductions in opioid requirements. In light of the ongoing public health concerns regarding long‐term opioid prescribing, these results are encouraging, though confirmation in larger and more diverse populations is warranted.

In this cohort, all seven recorded adverse events were hardware‐related (three lead migrations; four lead fractures or damage manifesting as high impedance). These occurred between 121 and 952 days post‐implant, often linked to external triggers such as falls or transfers. All were successfully managed with planned revision surgery, restoring therapy in every case. Two patients reached end of treatment: one due to progressive spondylolisthesis requiring spinal surgery, and another due to cutaneous lymphoma that resulted in skin erosion in proximity to the IPG pocket, necessitating explantation.

These findings align with previously published data. A systematic review and meta‐analysis by Vanloon et al. [31] reported rates of lead fracture and migration at approximately 6% each (95% CI: 2%–12%), supporting the consistency of our hardware‐related complication rates. Similarly, Chapman et al. [28] note that device‐related adverse event rates ranged from 3.1% to 36.8%, with neurologic events being rare, underscoring that hardware complications, whereas variable, are typically manageable. Manufacturer and user surveillance data also confirm that DRG‐S demonstrates a favorable safety profile comparable to or superior to that of conventional SCS [32]. Taken together, these findings underscore that DRG‐S is generally safe in long‐term use, with complications limited to hardware‐related issues that are typically correctable through revision procedures. The rare instances of end‐of‐treatment reflected comorbid conditions rather than inherent device failure, further supporting the durability and safety of DRG‐S in clinical practice.

Several methodological strengths enhance the credibility of our findings. Data collection was systematic, prospective, and supported by a digital platform with a high rate of follow‐up completion. The study also employed validated outcome measures across pain, function, mood, quality of life, and patient satisfaction, ensuring a comprehensive assessment of therapeutic impact. Furthermore, the medication analysis applied rigorous handling of missing data and standardized conversion factors for opioid dosing, which enhances interpretability.

Several limitations of this study must be acknowledged. Foremost, the cohort remains relatively small, and the number of patients available for analysis at 24 months was fewer than at 12 months. This reduction does not reflect attrition, but rather the fact that not all patients had yet reached 24 months of therapy at the time of analysis. As a result, statistical power was reduced and the risk of bias increased, highlighting the inherent challenges of long‐term real‐world follow‐up in clinical practice. The design, although based on prospectively collected registry data, also introduces potential confounding and selection biases. Finally, the absence of a control group precludes definitive attribution of observed benefits to DRG‐S alone, as natural history, regression to the mean, or placebo effects cannot be excluded.

Another limitation is reliance on patient‐reported medication data, which may be subject to recall bias or incomplete reporting. Although unclear entries were excluded by protocol, residual uncertainty about dosing patterns cannot be fully ruled out. Additionally, programming strategies evolved during the study, including the introduction of low‐frequency paradigms, which improve external validity but introduce heterogeneity that complicates interpretation.

Despite these caveats, the consistent benefits across multiple domains, robust opioid reduction, and acceptable safety profile collectively support the clinical value of DRG‐S for patients with refractory axial LBP. The results complement controlled trials and add important long‐term real‐world evidence.

Future research should prioritize multicentre, prospective studies with larger sample sizes and standardized protocols to confirm the durability and generalizability of these findings. Health‐economic analyses will also be essential to assess the cost‐effectiveness of DRG‐S, particularly in the context of reduced medication burden and potential improvements in productivity and quality of life. Further work is also warranted to better understand the mechanisms underlying DRG‐S, particularly its effects on central sensitization and maladaptive cognitive processes, as suggested by the marked improvements in PCS domains.

In summary, while our results must be interpreted cautiously, they suggest that bilateral T12 DRG‐S offers a durable, multidimensional benefit in selected patients with persistent spinal pain, including significant opioid‐sparing effects, improved function, and reduced catastrophic thinking. These findings highlight the potential of DRG‐S as a valuable component of multimodal care for chronic LBP.

## 5 Conclusion

This prospective registry analysis indicates that bilateral T12 DRG‐S provides durable, multidimensional benefits for patients with persistent spinal pain. Over 2 years, patients experienced significant reductions in pain, improved physical function, psychosocial health, and sleep, along with consistently high satisfaction. A marked decline in opioid use further underscores the potential of DRG‐S as an opioid‐sparing intervention. Safety was acceptable, with complications limited to hardware issues that were manageable with revision, and therapy discontinuation was rare and unrelated to device failure. Although confirmation in larger and controlled studies is warranted, these findings add important real‐world evidence supporting DRG‐S as a valuable treatment option for refractory axial LBP.

## Author Contributions

Dr. Pedram Tabatabaei and Dr. Johan Vänman have designed the study. Dr. Pedram Tabatabaei has conducted the study including patient recruitment and data collection. Dr. Pedram Tabatabaei and Dr. Johan Vänman are responsible for the data interpretation and statistical analysis of the data. However, all authors have had access to the study data. Dr. Pedram Tabatabaei has prepared the manuscript draft with important input from Dr. Maria Eriksson, Dr. Amar Awad, and Dr. Johan Vänman. All authors have approved the final manuscript.

## Funding

The authors have nothing to report.

## Ethics Statement

Data extraction from the Neuropain quality database was conducted under approval from the Swedish Ethical Review Authority (Dnr 2022‐05623‐01) and in accordance with applicable regulations and ethical standards.

## Consent

This study was approved by the Swedish Ethical Review Authority (Dnr 2022‐05623‐01); individual patient consent was not required because data were obtained from a quality registry. All patients are informed in advance about data collection and their rights in accordance with the EU GDPR and applicable Swedish legislation.

## Conflicts of Interest

Pedram Tabatabaei reports a relationship with Abbott Laboratories that includes speaking and lecture fees. There is no other conflicts of interest to report.

## Data Availability

The data that support the findings of this study are available on request from the corresponding author. The data are not publicly available due to privacy or ethical restrictions.
